# Therapeutic apheresis in kidney diseases: an updated review

**DOI:** 10.1080/0886022X.2022.2073892

**Published:** 2022-06-19

**Authors:** Yi-Yuan Chen, Xin Sun, Wei Huang, Fang-Fang He, Chun Zhang

**Affiliations:** Department of Nephrology, Union Hospital, Tongji Medical College, Huazhong University of Science and Technology, Wuhan, China

**Keywords:** Therapeutic apheresis, plasma exchange, double-filtration plasmapheresis, immunoadsorption, kidney transplantation

## Abstract

Besides conventional medical therapies, therapeutic apheresis has become an important adjunctive or alternative therapeutic option to immunosuppressive agents for primary or secondary kidney diseases and kidney transplantation. The available therapeutic apheresis techniques used in kidney diseases, including plasma exchange, double-filtration plasmapheresis, immunoadsorption, and low-density lipoprotein apheresis. Plasma exchange is still the leading extracorporeal therapy. Recently, growing evidence supports the potential benefits of double-filtration plasmapheresis and immunoadsorption for more specific and effective clearance of pathogenic antibodies with fewer side effects. However, more randomized controlled trials are still needed. Low-density lipoprotein apheresis is also an important supplementary therapy used in patients with recurrent focal segmental glomerulosclerosis. This review collects the latest evidence from recent studies, focuses on the specific advantages and disadvantages of these techniques, and compares the discrepancy among them to determine the optimal therapeutic regimens for certain kidney diseases.

## Graphical Abstract

**Figure:**
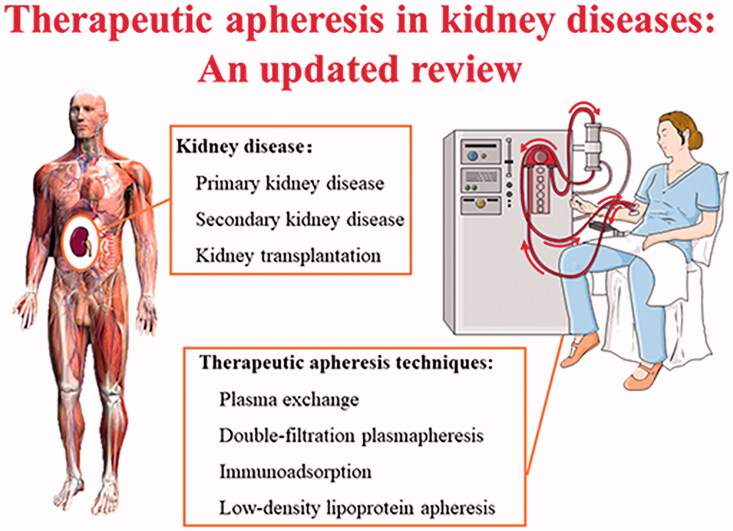

## Introduction

The terms therapeutic apheresis (TA) and therapeutic plasma exchange (TPE) are used to describe the process of removing pathological substances, such as cells, pathogenic antibodies, immune complexes, and inflammatory mediators, from the patient by an extracorporeal apheresis system to purify the blood and treat diseases. The widely used TA techniques include plasma exchange (PE), double-filtration plasmapheresis (DFPP), immunoadsorption (IAS), and low-density lipoprotein apheresis (LDL-A) (Table 1).

**Table 1.** Comparison of four therapeutic apheresis techniques.

	PE	DFPP	IAS	LDL-A
Selectivity	Non-selective	Semi-selective	Semi-selective	Semi-selective
Plasma processing volume	1–1.5 times (very limited)	1–2 times (limited)	2–3 times (unlimited theoretically)	2–3 times (unlimited theoretically)
Substitution solution	Crystalloid/colloid (HSA or FFP)	Little HSA or saline	No substitution solution	No substitution solution
Removal of protein	Remove all plasma components	Remove macromolecules	Remove pathogenic factors selectively (predominantly immunoglobulins)	Remove LDL and other lipoproteins

PE: Plasma exchange; DFPP: double-filtration plasmapheresis; IAS: immunosorption; LDL-A: low-density lipoprotein apheresis; HSA: human serum albumin; FFP: fresh frozen plasma.

As an adjunctive therapeutic option, TA is widely used in the treatment of primary or secondary kidney diseases and kidney transplantation (KT) (Table 2). Recommendation grades and indication categories of TA in the treatment of kidney diseases have been updated in 2019 American Society for Apheresis (ASFA) guidelines (Table 3) [1]. However, whether one technique is a better choice than another is still uncertain owing to a lack of randomized controlled trials. This review will focus on the available evidence and compare the differences among various TA techniques in treating these diseases to determine the optimal therapeutic method for particular kidney diseases.

**Table 2.** Indications for therapeutic apheresis in diseases involved kidney and their pathogenic factors.

Medical disciplines	Diseases	Pathogenic factors
Primary kidney diseases	FSGS	Circulatory permeability factors
	MN	PLA2R Ab and THSD7A Ab
	Anti-GBM glomerulonephritis (Goodpasture’s syndrome)	Anti-GBM Ab
Secondary kidney diseases	ANCA-associated vessel vasculitis	Anti-MPO or anti-PR3 Ab
	TTP	ADAMTS-13 Ab, ICs
	aHUS	Complement regulatory components or autoantibodies
	SLE	Anti-dsDNA Ab, anti-nuclear Ab, ICs
KT	ABO-incompatible KT	Blood group isoagglutinins
	HLA-incompatible KT	HLA and non-HLA alloantibodies
	Ab-mediated allograft rejection	HLA and non-HLA alloantibodies

FSGS: Focal segmental glomerulosclerosis; MN: membranous nephropathy; PLA2R: M-type phospholipase A2 receptor; THSD7A: thrombospondin type 1 domain-containing protein 7 A, Ab: antibody; GBM: glomerular basement membrane; ANCA: antineutrophil cytoplasmic antibodies; MPO: myeloperoxidase; PR3: proteinase 3; TTP: thrombotic thrombocytopenic purpura; ADAMTS-13: a disintegrin-like and metalloprotease with thrombospondin type 1 motifs-13; ICs: immune complexes; aHUS: atypical hemolytic uremic syndrome; SLE: systemic lupus erythematosus; KT: kidney transplantation; HLA: anti-human leukocyte antigen.

**Table 3.** Therapeutic apheresis for the treatment of kidney diseases: recommendation grades and indication categories in 2019 American Society for Apheresis guidelines [1]. ABO incompatibleII

Disease	Indication	Apheresis	Category	Recommendation grade	Technical notes
FSGS	Recurrent in KT	PE/IAS	I	Grade 1B	Volume treated: TPE, LA, or IA with single use adsorbers: 1.0–1.5 TPV; IA with regenerative adsorbers: 2–3 TPV.Frequency: Daily or every other day at initiation of treatment. Subsequent frequency and duration based on patient response.
	Recurrent in KT/Steroid resistant in native kidney	LDL-A	II	Grade 2C	
	Steroid resistant in native kidney	PE	III	Grade 2C	
Anti-GBM glomerulonephritis	DAH	PE	I	Grade 1C	Volume treated: 1–1.5 TPVFrequency: daily or every other day for 14 days or until anti-GBM undetectable
	Dialysis-independence	PE	I	Grade 1B	
	Dialysis-dependence (Cr > 5.7mg/dl)	PE	III	Grade 2B	
ANCA-associated disease	MPA/GPA/RLV				Volume treated: 1–1.5 TPVFrequency: daily in DAH, typically every other day in absence of DAH
	RPGN, Cr ≥ 5.7mg/dl	PE	II	Grade 1B	
	RPGN, Cr < 5.7 mg/dl	PE	IIII	Grade 2C	
	DAH	PE	I	Grade 1C	
	EGPA	PE	III	Grade 2C	
SLE	Severe complications	PE	II	Grade 2C	Volume treated: 1–1.5 TPVFrequency: LN or DAH: daily or every other day; Other severe complications: 1–3 times per week. Typically course of 3–6 PE is enough to see response
TMA	TTP	PE	I	Grade 1A	Volume treated: 1–1.5 TPVFrequency: daily until platelets >150K and LDH near normal for 2–3 consecutive days, taper vs abrupt discontinuation practices vary
	STEC-HUS	PE/IAS	III	Grade 2C	Volume treated: 1–1.5 TPVFrequency: daily until improvement, no standardized approach exists
	aHUS				Volume treated: 1–1.5 TPVFrequency: daily until clinical response (complement mediated), daily or every other day for coagulation mediated TMA
	Factor H autoantibody	PE	I	Grade 2C	
	CF gene mutations	PE	III	Grade 2C	
KT					
ABO incompatible	Desensitization	PE/IAS	I	Grade 1B	Volume treated: 1 - 1.5 TPV Frequency: daily or every other day. antibody titer is less than critical threshold prior to before KT
	AMR	PE/IAS	II	Grade 1B	
ABO compatible	Desensitization	PE/IAS	I	Grade 1B	Volume treated: 1–1.5 TPVFrequency: usually 5 or 6, daily or every other day
	AMR	PE/IAS	I	Grade 1B	

FSGS: Focal segmental glomerulosclerosis; KT: kidney transplantation; PE: plasma exchange; IAS: immunoadsorption; LDL-A: low-density lipoprotein apheresis; GBM: glomerular basement membrane; DAH: diffuse alveolar hemorrhage; ANCA: antineutrophil cytoplasmic antibodies; MPA: microscopic polyangiitis; GPA: granulomatosis with polyangiitis; RLV: renal-limited vasculitis; EGPA: eosinophilic granulomatosis with polyangiitis; RPGN: rapidly progressive glomerulonephritis; SLE: systemic lupus erythematosus; TMA: thrombotic microangiopathy; TTP: thrombotic thrombocytopenic purpura; STEC-HUS: shiga toxin-mediated hemolytic syndrome; aHUS: atypical hemolytic uremic syndrome; CF: complement factor; AMR: antibody-mediated rejection; TPV: total plasma volume.

## Modalities of TA

### Methods of plasma separation: centrifugation or filtration

All four TA techniques separate the plasma first. Centrifugation and filtration are the most common plasma-separation techniques [2]. Plasma separation by centrifugation (cTPE) is achieved using a rapidly rotating centrifuge in an automated system designed to non-selectively separate plasma from the rest of the blood based on density and centrifugal force. Erythrocytes are removed first, followed by leukocytes and platelets. There is no upper limit to the molecular weight of proteins removed by this method. The advantages of cTPE include its low cost, low required anticoagulant and blood flow rate, which are important for patients in critical conditions. Despite the improvement of this technique, obtained plasma still has minor platelet contamination, which might interfere with the adsorption process. Plasma separation by filtration (mTPE) is achieved by a hollow fiber plasma filter whose highly permeable membrane is sufficient to separate plasma from blood components based on pore size. Membrane plasma separation has the advantage of achieving platelet-free plasma in the apheresis process. However, some large immune complexes and cryoglobulins may not be adequately cleared [3]. In a randomized prospective crossover study comparing cTPE and mTPE, cTPE had a significantly higher efficiency in removing plasma with a shorter treatment time [4]. What’s more, compared to cTPE, mTPE cannot separate cellular components. It is used in plasma separation only but not used for cellular apheresis procedures, such as red blood cell exchange, leukapheresis, platelet removal, and stem cell harvesting.

### Choice of plasma processing volume and intervention frequency

The choice of plasma processing volume and intervention frequency depends on different clinical situations and how many of the pathogenic substances need to be removed from the body. Plasma processing volume is calculated based on the patient’s body mass and is generally 1–1.5 times the total plasma value (TPV) in PE, whereas the plasma processing volume is theoretically unlimited in IAS. In acute or critical clinical situations, the therapy is performed every day or every other day. According to the clinical results, the interval between two sessions is gradually extended. For maintenance therapy, a frequency of two to four times per month may be sufficient for patients in a relatively stable stage [5]. The frequency and duration of TA techniques used in different diseases are summarized in Table 3 according to ASFA guidelines.

### Advantages and disadvantages of different TA techniques

**Figure 1.**
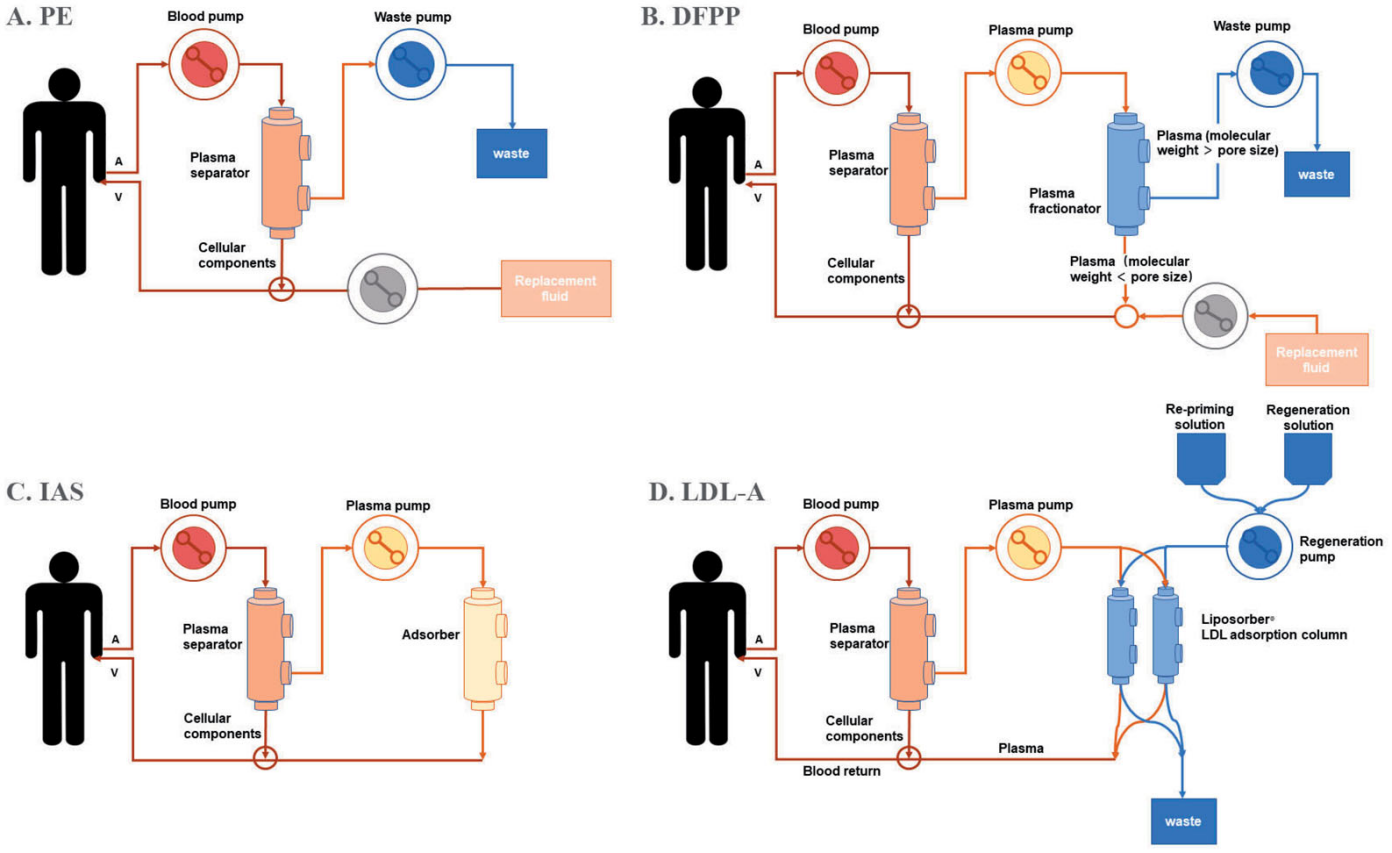
Schematic diagram of different therapeutic apheresis techniques. (A) In plasma exchange (PE), the separated plasma is discarded, while separated blood cells are returned to the body together with replacement fluids like human albumin or fresh frozen plasma. (B) In double filtration plasmapheresis (DFPP), the separated plasma passes through the second filter called ‘plasma fractionator’ with different pore size. Macromolecules larger than the pore size of the fractionator is discarded, while smaller molecules, such as small-molecular-mass IgG and albumin are returned to circulation together with replacement fluids. (C) In immunoadsorption (IAS), the separated plasma is taken to an adsorber unit, where specific pathogenic factors are specifically removed. Finally, the ‘purified’ plasma is returned to the body together with the separated blood cells. (D) In low-density lipoprotein apheresis (LDL-A), cholesterol particles including LDL, VLDL and Lp(a) in separated plasma are biding to dextran sulfate on the basis of electrical charge. Other important blood substances are not removed and returned to the patient.

PE is a therapeutic procedure in which plasma is first separated from other components of blood and then discarded and replaced with substitute fluid (Figure 1(A)). In this procedure, PE non-selectively removes all substances, such as pathologic antibodies, immune complexes, inflammatory mediators, albumin, and other useful components in the plasma. Therefore, a large volume of replacement fluid collected from healthy donors or consisting of manufactured human serum albumin or immunoglobulins needs to be reinfused into the patient’s circulation to avoid significant hypovolemia. This process increases the risk of transfusion-related infections or allergic reactions. However, PE also has beneficial effects for infusing useful plasma components, such as a disintegrin-like and metalloprotease with thrombospondin type-1 motifs-13 (ADAMTS-13) in thrombotic thrombocytopenic purpura (TTP).

DFPP is another therapeutic procedure, which uses two plasma filters with different pore sizes to selectively remove macromolecules, especially immunoglobulins (Figure 1(B)). The first step is to separate plasma from cellular components with the first filter, called the plasma separator. Then the separated plasma passes through the second filter, called the plasma fractionator, which blocks macromolecules larger than the pore size of the fractionator, whereas smaller molecules, such as small-molecular-mass IgG and albumin, filter through the fractionator and return to circulation. The volume of the replacement fluid needed is smaller than PE. By choosing filters with the optimal pore size, pathogenic substances can be selectively removed from IgG to low-density lipoprotein cholesterol (LDL-C). The disadvantage of DFPP is that the plasma fractionator cannot remove small-molecular-mass IgG and substances smaller than albumin. Numerous studies have shown that DFPP is a promising therapy in metabolic, rheumatic, and kidney diseases [6].

IAS is a new technique developed on the basis of PE. It has a high affinity for immune complexes through IAS columns to remove pathogenic factors selectively or specifically (Figure 1(C)). First, plasma and corpuscular elements are separated. Then, the plasma passes through different columns with varied degrees of affinity to circulatory immunoglobulins with or without antigen specificity. Finally, the purified plasma is returned to the patient's body. Compared with PE, IAS has higher selectivity for the removal of pathogenic factors in plasma. Theoretically, IAS does not need replacement fluid, such as FFP or albumin, thus avoiding all kinds of adverse effects caused by plasma infusion, such as infection or allergic reactions. In addition, the volume of processed plasma is theoretically unlimited [7].

LDL-A is a novel technique used for the treatment of nephrotic syndrome (NS), especially focal segmental glomerulosclerosis (FSGS), which selectively removes lipoprotein particles from the blood with the reinfusion of the remaining components (Figure 1(D)) [8]. There are four different techniques of LDL apheresis: IAS, dextran sulfate cellulose adsorption, heparin extracorporeal LDL precipitation, and direct adsorption of lipoprotein using hemoperfusion. All of these techniques are used for familial hypercholesterolemia, but only dextran sulfate cellulose adsorption can be used for drug-resistant NS [8]. The machine first separates plasma from the rest of the blood, and then, the Liposorber filter removes the LDL, very-low-density lipoproteins, Lipoprotein (a), and triglycerides from the plasma. Finally, the hemocytes and purified plasma are returned to the patients. Low blood pressure is the most common adverse effect associated with LDL-A.

## TA in primary kidney diseases

### Focal segmental glomerulosclerosis

To some extent, FSGS is a set of clinical-pathological syndromes, rather than a single disease. Circulatory permeability factors in plasma have been suggested as the pathogenic factors of FSGS, which target podocytes and damage the glomerular filtration barrier [9]. Typically, for the treatment of primary FSGS, corticosteroid is considered as the first-line therapy, and immunosuppressive agents such as cyclophosphamide (CYC), calcineurin inhibitors (CNI), rituximab (RTX), or mycophenolate mofetil (MMF) are recommended in patients with steroid-dependent or steroid-resistant conditions [10–13]. However, partial remission is still more common than complete remission, and these drugs can cause severe complications [14]. Currently, extracorporeal therapies, such as PE, IAS, and LDL-A, are reserved for patients with drug-resistant or drug-based treatment failure [15–17].

Several studies indicated that PE was beneficial for patients with FSGS. The proteinuria and renal function of patients with steroid-resistant NS was improved with PE [15,18]. In one report, four patients with FSGS in the native kidney were treated with IAS. Only one patient achieved partial remission after treatment [16]. Neither the proteinuria nor the renal function of the other three patients changed, and renal dysfunction even progressed, which might be associated with the negative permeability factors and the stage of nephropathy. According to ASFA guidelines, PE is only used when other prior therapies have failed [1]. More studies need to be conducted to determine whether IAS is an effective alternative therapy.

Persistent hyperlipidemia in patients with FSGS is nephrotoxic and can lead to chronic progressive glomerular and tubulointerstitial injuries. LDL-A might be a promising supplementary treatment. In the Prospective Observational Survey on the Long-Term Effects of LDL Apheresis on Drug-Resistant Nephrotic Syndrome, the efficacy of LDL-A for FSGS was assessed [17]. For 28 patients with refractory FSGS, nearly half of the patients (42.9%) were in remission within two years after LDL-A treatment and achieved a favorable outcome. LDL-A has been shown to reduce proteinuria in FSGS patients and improve the response rate to steroids and immunosuppressive therapies. The possible reason for this might be that reduced LDL levels alleviated the lipotoxic effect on podocyte function and improved intracellular drug transport.

In conclusion, extracorporeal therapies might be important complementary options in the treatment of primary refractory FSGS in the native kidney. The applications of these techniques are still rare in native kidney diseases, and more studies are needed to verify their efficacy and safety.

### Membranous nephropathy

The pathogenesis of membranous nephropathy (MN) has been confirmed since the discovery of autoantibodies against podocyte M-type phospholipase A2 receptor (PLA2R) and thrombospondin type 1 domain-containing protein 7 A (THSD7A) [19,20]. Other autoantigens, such as neural epidermal growth factor-like 1 protein (NELL-1), exostosin 1 and 2, have also been discovered recently [21,22]. The titers of autoantibodies are closely related to the prognosis of MN. Despite advances in understanding the pathogenesis, treatment regimens remain intractable. For severe patients, the standard treatment is still a six-month regimen of rotating high-dose steroids and immunosuppression, such as the modified Ponticelli regimen (steroids and CYC) or modified Cattran regimen (steroids and Cyclosporine A) [23–25]. But these regimens are always accompanied by significant adverse effects, including infection, osteoporosis, diabetes mellitus, weight gain, hemorrhagic cystitis, infertility, or cancer [26]. The discovery of nephritogenic autoantibodies provides a new therapy choice that targets B-cell lineages to prevent antibody production [27].

As an antibody-mediated disease, extracorporeal therapy seems to be a promising option. For patients treated with immunosuppression who still do not achieve remission or relapse after remission, a rescue therapy regimen with PE, intravenous immunoglobulins (IVIGs), and rituximab might be beneficial [27]. In this retrospective study, nine of 10 patients who had been resistant to all conventional regimens achieved partial remission with a rescue therapy regimen. The mean time in which they achieved partial remission was 2.1 ± 0.5 months, which was significantly shorter compared to the three other regimens (rituximab only, modified Ponticelli regimen, and modified Cattran regimen). In addition, patients with PLA2R-negative MN also responded to this therapy. PE seems to be a treatment option for patients who are refractory to conventional therapies, but further and larger retrospective studies are needed to evaluate the efficacy of PE.

IAS was first reported as a treatment option in 1999 [28]. It reduced proteinuria in different nephrotic syndromes, including four patients with MN. A prospective phase II clinical trial was carried out to assess the safety and efficacy of IAS in MN [29]. Patients with serum anti-PLA2R antibody titer levels above 170 μmol/L underwent five consecutive daily sessions of IAS. A regenerative GAM-146-peptide-coated (Gifu anaerobic medium, Globaffin^®^) column was chosen, which can specifically remove IgG, particularly IgG1, IgG2, and IgG4 [30]. The results showed that IAS reduced the titers of PLA2R antibodies, but regenerated antibodies may limit the efficacy of solitary IAS. In patients with PLA2R-negative and THSD7A-positive MN, IAS can also reduce proteinuria by reducing THSD7A antibody levels. However, proteinuria relapses and autoantibodies regenerate when IAS is discontinued [31]. Therefore, IAS should be an adjunctive option for severe cases.

PE and IAS can be useful tools in the treatment of severe antibody-positive MN. But for long-term remission of MN, immunosuppressive therapies are still the leading therapy. IAS or PE might only be used to remove autoantibodies in patients with severe proteinuria accompanied by complications such as serious infections.

### Anti-GBM glomerulonephritis (Goodpasture syndrome)

Anti-glomerular basement membrane (anti-GBM) glomerulonephritis is a rare but potentially life-threatening autoimmune disease characterized by rapidly progressive glomerulonephritis (RPGN) with or without diffuse alveolar hemorrhage (DAH). The occurrence of both manifestations is known as Goodpasture syndrome, which has a high mortality rate [32]. Anti-GBM disease is mediated by circulatory anti-GBM antibodies targeting an epitope on the non-collagenous domain of the α3 chain of collagen type IV, which activating the complement cascade, results in tissue injury [33]. Antineutrophil cytoplasm antibodies (ANCA) can also be detected in 25% of patients [34]. The titers of these antibodies are associated with disease severity. Because the antibody is pathogenic, clearance of these antibodies is the most effective treatment.

Current standard treatments include combining PE with immunosuppressants such as CYC and corticosteroids. While PE removes the circulatory antibodies, immunosuppressants inhibit the formation of new antibodies. Before the application of PE, the mortality and ESRD caused by Goodpasture syndrome were extremely high. In several nonrandomized trials, PE was found to be effective in reducing anti-GBM antibodies, improving renal function, and controlling intra-alveolar bleeding [35]. For patients with serum creatinine <5.7 mg/dl and DAH, the renal function might recover after treatment, but for patients with serum creatinine >5.7 mg/dl or dialysis dependence, glomerular injury and kidney function are usually irreversible [1,36]. Therefore, PE should be implemented early before the disease develops to ESRD.

IAS is an alternative therapy for removing circulatory factors, but only a few cases reported using IAS in anti-GBM disease [37,38]. In a study of 10 patients with anti-GBM disease treated with IAS, anti-GBM antibodies were reduced to negative levels in patients after two through nine sessions of IAS treatment, and dialysis dependency was successfully reversed in three of six patients [39]. Owing to the high response, IAS should be considered as a valuable treatment option for anti-GBM disease, especially in patients with a high percentage of crescents and dialysis dependence. Another study showed that the efficacy of clearing anti-GBM antibodies, renal survival, and patient survival were similar between the DFPP and IAS groups [40]. Compared to PE, DFPP and IAS are commonly considered more expensive, but for patients who need intensive TA therapy (15 or more treatments), total costs might be lower than PE because of reusable adsorbers [39].

## TA in secondary kidney diseases

### Antineutrophil cytoplasm antibodies-associated vasculitis

ANCA-associated vasculitis (AAV) includes microscopic polyangiitis (MPA), granulomatosis with polyangiitis (GPA, previously Wegener’s granulomatosis), and eosinophilic granulomatosis with polyangiitis (EGPA, previously Churg-Strauss syndrome) [41]. AAV is characterized by the formation of necrotizing inflammation and fibrinoid necrosis in small vessels [41], and the development of autoantibodies to the neutrophil cytoplasmic antigens with specificity for proteinase 3(PR3-ANCA) or myeloperoxidase (MPO-ANCA) [42]. Many organs can be affected, especially the kidney; lung; ear-nose-throat (ENT); joint; skin; and nerve system.

For decades, glucocorticoids and immunosuppressants (CYC or Rituximab) were considered as the standard treatment for AAV. For patients with severe clinical symptoms such as DAH or RPGN, PE is used as the first-line therapy. Nevertheless, the benefit of PE is still in doubt. PE is recommended based on the Methylprednisolone versus Plasma Exchange (MEPEX) trial (the largest long-term follow-up study of PE in AAV), which suggested using either PE or high-dosage methylprednisolone as adjunctive therapy [43]. In this study, 137 patients with severe renal diseases (serum creatinine >5.7 mg/dl or on dialysis) received intravenous methylprednisolone (IVMP) or PE added to oral CYC and glucocorticoids. A higher rate of renal recovery and dialysis independence was achieved in the PE group than in the IVMP group. But after four years, PE showed no benefits to overall survival, renal survival, or relapse rate [44]. Moreover, the MEPEX trial showed that patients without scarring on their renal biopsy are most likely benefit from PE [43]. However, patients with ESRD may not benefit from PE and may even suffer serious adverse effects from PE. PE may increase the risk of death in patients with DAH because the infection is the most common cause of death. Whether PE should be recommended for patients with severe kidney dysfunction or DAH is controversial. Recently, the Plasma Exchange and Glucocorticoids for Treatment of ANCA-Associated Vasculitis (PEXIVAS) trial (*n* = 704) were conducted, and the inclusion criteria was broader, including patients with kidney dysfunction or DAH [45]. The purpose of this trial was to evaluate the effect of PE in severe AAV. PE showed a transient benefit but did not reduce the mortality or incidence of ESRD in further follow-ups for two to seven years. There was no statistical significance between the subgroups of patients with creatinine >5.7 mg/dl or DAH. A meta-analysis of trials, including MEPEX and PEXIVAS, showed PE significantly decreased overall incidence of ESRD in a short time, but it may not have a positive effect on long-term prevention of the development of ESRD [46]. Despite the negative findings, PE was still supported by many physicians. In PEXIVAS, renal biopsies were not required, limiting the ability to determine whether PE may be beneficial in subgroups of patients based on histological changes. The controversy in different studies may also be due to the difference in eligibility, end points, and trial duration. The Kidney Disease/Improving Global Outcomes (KDIGO) 2021 clinical practice guidelines do not recommend the routine use of PE for patients with eGFR <50 mL/min per 1.73 m^2^ but state it can be considered for patients with creatinine >5.7 mg/dl requiring dialysis or with rapidly increasing creatinine, and in patients with DAH who have hypoxemia. For patients with an overlapping syndrome of AAV and anti-GBM, PE should be added [47]. These recommendations are dependent on the MEPEX trial and a meta-analysis, and maintenance therapy should be continued after PE.

The application of IAS in AAV is relatively rare. Only a few studies reported the efficacy of IAS in autoantibody clearance in AAV with favorable outcomes, but no evidence showed that IAS was superior to PE [48]. Ten patients with RPGN (five with MPA, two with GPA, and three with SLE) on dialysis were treated with IAS, prednisolone, and CYC [48]. Renal function was recovered within an average of 6.7 days in nine of 10 patients, whereas with PE, the average recovery time was 11.5 days. Only three patients had significant regeneration of antibodies at 1-month post-IAS. In general, IAS or PE combined with glucocorticoids and CYC may be valuable for the treatment of RPGN, but more studies are needed to confirm their efficacy and benefit.

### Thrombotic microangiopathy

Thrombotic microangiopathy (TMA) involved in kidney is the most common indication of PE [49]. TMA can be classified as thrombotic thrombocytopenic purpura (TTP) for predominant neurologic involvement and hemolytic uremic syndrome (HUS) for kidney dominant diseases [50]. TTP and HUS have similar clinical manifestations, such as microangiopathic hemolytic anemia, consumptive thrombocytopenia, and microvascular thrombosis, but the treatment protocols are different.

#### Thrombotic thrombocytopenic purpura

The pathophysiology of TTP is related to deficiency of von Willebrand factor (vWF)-cleaving metalloprotease known as ADAMTS13 [51]. TTP is caused by a congenital deficiency (hereditary/congenital TTP) or inhibition by autoantibodies (acquired TTP).

Acquired TTP is the most prevalent form of the disease (>95%), which is related to the formation of autoantibodies against ADAMTS13 [52]. PE with FFP replacement is the foundation of first-line therapy, which not only can delete vWF multimers and anti-ADAMTS13 antibodies, but also can replenish normal vWF and ADAMTS13 [53]. Mortality is reduced from 90% to <20% with PE [54]. PE should be initiated immediately once the diagnosis is recognized. Plasma infusion (PI) alone can also improve ADAMTS13 activity, but the inhibitors will remain. Evidence shows that PE is superior to PI in treating TTP [55]. Immunosuppressive therapies such as glucocorticoids and rituximab are cornerstone regimens for acquired TTP [56]. However, repurposed drugs and novel agents are becoming new options [57]. Caplacizumab is a novel agent applied to TTP, which can effectively block the end organ damage caused by TTP [58]. With the high efficiency of this agent, the treatment of acute TTP may enter an era without obligatory PE [59].

Congenital TTP is rare and associated with somatic mutations resulting in severe ADAMT13 deficiency, and no ADAMT13 autoantibody inhibitors are involved in congenital TTP [51]. PI alone is sufficient. The frequency of PE depends on clinical symptoms and platelet counts [60].

The application of IAS in TTP is extremely rare, and only a few cases have been reported. In these cases, patients had an initial response to intensive PE with FFP, but the attempt to decrease the frequency of PE resulted in decreased platelet counts, increased lactate dehydrogenase (LDH), and neurological deterioration. IAS was used in this situation and patients’ platelet counts, LDH, and neurological states normalized [61,62]. IAS seems to be more effective than PE in patients with TTP who have inhibitors of ADAMTS13.

In conclusion, once TTP is diagnosed, PE should be initiated, which could significantly improve patients’ prognoses. For patients with refractory TTP, rituximab, immunosuppressive agents, and novel agents can be used. More prospective studies are needed to verify whether IAS could be a treatment option.

#### Hemolytic uremic syndrome

Typical HUS is characterized by microangiopathic hemolytic anemia, thrombocytopenia, and severe kidney injury. The most common cause of HUS is infection by shiga toxin-producing bacteria, mainly Escherichia coli O157:H7 (STEC) [63]. Most infected children develop renal insufficiency, and two-thirds of them need dialysis. Thus, typical HUS is treated with supportive care, including appropriate fluid, electrolyte management, and hemodialysis when clinically indicated. In a prospective study, 12 patients with STEC-HUS (O104:H4) showed no response to PE or complement-blocking antibodies (eculizumab) and developed severe neurological complications. IAS was then successfully used as rescue therapy, with all 12 patients surviving, and 10 of them achieving complete neurological and renal function recovery [64].

Atypical HUS (aHUS), also known as complement-mediated TMA, is caused by dysregulation of the complement system, leading to TMA and kidney injury [65]. Mutations in complement factor H (CFH), complement factor I, complement 3, membrane cofactor protein, and thrombomodulin are reported [65–67]. Moreover, anti-CFH autoantibodies are detected in up to 70% of patients with aHUS [68]. The complement system may be activated by infection, pregnancy, autoimmune disease, transplantation, or drugs in the presence of these mutations [69]. Eculizumab is an inhibitor to complement 5, blocking the activation of the complement system; its efficacy and safety have been proven in a large trial [70]. Historically, PE is recommended as the initial treatment for acute HUS before aHUS is diagnosed. Once other causes of TMA are excluded, eculizumab should be used. The prognosis of PE alone is generally poor. After long-term follow-up, up to 79% of patients have permanently decreased kidney function or ESRD [71]. In a retrospective study with 31 patients, patients treated with eculizumab accompanied by PE/PI showed better efficacy compared to those treated with PE/PI alone [72]. PE or PI is still recommended as a supportive and alternative therapy when eculizumab is not available.

### Systemic lupus erythematosus

Systemic lupus erythematosus (SLE) is a common autoimmune disease, which can cause immune complex deposition and inflammation of multiple organs. Pathogenic autoantibodies, such as antinuclear antibody, anti-double stranded DNA antibody, and anti-Smith antibody, play an important role in the pathogenesis of SLE and indicate disease activity [73,74]. Inhibiting or reducing the production of these autoantibodies can prevent severe organ damage. Lupus nephritis (LN) is the most common serious organ manifestation of SLE.

Glucocorticoids and immunosuppressive therapies are the most traditional strategies for SLE. Moreover, due to disease progression and the serious side effects of the long-term use of glucocorticoids, the clinical prognosis is not as good as expected [75]. Refractory SLE is still a therapeutic challenge. Extracorporeal therapies including PE and IAS have been used in refractory cases or when conventional immunosuppressive procedures are contraindicated.

Since PE was first reported to be useful in SLE in 1976 [76], this therapy has been widely used. However, in a large randomized controlled trial initiated in 1982, PE combined with standard of care (SOC) showed no difference in clinical outcomes compared to SOC alone [77]. In addition, when combined with intravenous CYC, the disease may get worse because of fatal bacterial and viral infections [78]. PE is not mentioned as an induction or maintenance therapy [79], but for SLE with life-threatening situations (e.g., DAH, TMA, hyperviscosity, cryoglobulinemia, antiphospholipid syndrome, pregnancy), rapidly removing autoantibodies and circulatory immune complexes with PE may still be an important therapy [80].

At present, many researchers have applied IAS to treat severe and refractory SLE and have achieved good clinical results. Several studies showed that IAS with different columns significantly decreased disease activity, and improved clinical outcomes [81]. Not surprisingly, high-affinity columns like Ig-Therasorb column have superior effects in removing antibodies [82,83]. Stummvoll et al. found that in 16 patients with severe SLE and LN in whom CYC was contraindicated or failed to prevent disease progression, IAS was found to significantly reduce proteinuria, alleviate disease activity, and lower glucocorticoid dosages within three months of therapy [82,84]. Not only short-term efficacy but also long-term efficacy is considered. Patients who had not achieved remission with respect to proteinuria were observed to further decline beyond the original decline after the first-year follow-up [85]. In this 10-year study with up to 324 IAS sessions per patient, IAS was safely applied without infections, malignancies, or other unwanted reactions. In a retrospective study, HA280 and DNA280 IAS columns were found to effectively remove pathogenic factors, thus controlling the activities of severe SLE. The application of HA280 column is a more cost-effective therapy for severe SLE than the application of DNA280 column [86].

In conclusion, extracorporeal therapy is a reasonable choice in patients with severe manifestations, especially when conventional strategies fail or are contraindicated. IAS is the preferred option compared to PE due to the low frequency of side effects. However, the ideal duration and the choice of columns used for treatment have not been defined.

## TA in kidney transplantation

### Desensitization in ABO blood group incompatible kidney transplantation

KT provides a substantial result in terms of survival and quality of life compared to hemodialysis or peritoneal dialysis for patients with ESRD, but the graft shortage remains a problem [87,88]. ABO blood group incompatibility was once considered as an absolute contraindication to KT, using preconditioning therapy is a viable option. The reduction of the concentration of antibodies against A and B blood group antigens prevents antibody-mediated rejection (ABMR) [89]. Beside conventional immunosuppression, the current preconditioning therapies are B-cell depleting therapies and TA [90]. Splenectomy used to be the mandatory intervention for ABO blood group incompatible kidney transplantation (ABOi-KT), but it was recently replaced by rituximab due to the surgical risk and increased risk of sepsis [91,92]. ASFA guidelines strongly suggest PE or IAS as desensitization protocols in ABOi-KT [1].

There is significant supportive evidence that PE can facilitate ABOi-KT. Particularly, a study from Japan (>2000 cases) has shown that five- and 10-year graft survival was 95% and 90%, respectively [93]. ABOi-KT achieved excellent long-term (>20 years) outcomes [94]. There were no statistically significant differences in graft survival rates between ABOi-KT after desensitization and ABO blood group compatible KT [95].

The disadvantage of using PE is the loss of physiological proteins with a treating dose limited to 1.0–1.5 times the TPV [1]. In a study of patients from Indian, the treatment regimen was started with rituximab about seven to 14 days before IAS, and the aim of the titer level of antibodies was eight or lower given that high titer levels are always accompanied by higher rates of ABMR [96]. IAS successfully achieved the target ABO antibody titer level in all patients, and kidney function performed well after KT.

Most transplant centers use the same desensitization strategies, regardless of the basal titers of the ABO blood group antibodies. In a systematic review, PE and IAS were used in an average of four sessions before KT [97]. However, in a single-center study from the United Kingdom, they assumed that a regimen could be tailored according to the initial ABO blood group antibody titers, with patients with lower titers requiring less desensitization. In this study, DFPP was used in patients with titer levels between 16 and 64, whereas antigen-specific IAS (glycosorb-ABO IAS columns) was performed in patients with titer levels >64 [98]. The reason is that patients with higher titer levels need more cycles of antibody removal, and IAS is more expensive and has a lower bleeding risk than serial DFPP [99]. The patients with initial antibody titer levels ≤8 who receive no TA techniques pretransplant or posttransplant did not undergo ABMR or graft loss. Although this study included only a small group of patients, we consider it is a feasible strategy to minimize desensitization to avoid certain complications and reduce medical expenses.

### Desensitization in HLA-incompatible kidney transplantation

Patients’ sensitization to anti-human leukocyte antigen (HLA) is another immunological barrier. Preexisting donor-specific anti-HLA antibodies (DSA) come from sensitizing exposures, such as transfusion, pregnancy, or previous transplantation. About 30% of candidates have detectable DSA, and half of them are sensitized to 80% of potential donors [100]. In general, KT is best performed in the absence of DSA, but for these immunologically disadvantaged patients, desensitization before KT is a relatively good option [101]. HLA desensitization significantly improves access to KT by eliminating or reducing HLA antibody levels to obtain a negative cytotoxic crossmatch. Current desensitization protocols include TA techniques (including PE, DFPP, and IAS), IVIG, and B-cell-depleting agents [102].

In the early 1970s, PE was used to remove DSA in acute humoral rejection [103]. PE achieved a negative crossmatch in desensitization compared to IVIGs [104], but evidence showed that DSA had rebounded when PE was stopped [105]. It was a challenge to maintain low or nonexistent anti-HLA antibody levels.

IAS was first successfully used for desensitization in 1989 [106]. In 2007, a study showed the efficacy of IAS to eliminate DSA in active ABMR post KT [107]. Rapid rebound of DSA soon after KT can be solved by repeated IAS treatment. Besides, only a few studies with small populations compared the efficacy of different TA techniques in desensitization. Forty-five patients who underwent TA for HLA-incompatible (HLAi) KT in a single center were analyzed [108]. It was shown that TA protocol with rituximab and a standard immunosuppressive regimen are effective for patients in the setting of HLAi-KT. IAS is more effective to remove IgG and anti-HLA antibodies, especially for class II DSA and has better tolerance than DFPP.

### Antibody-mediated allograft rejection

ABMR is widely recognized as the main reason for graft loss after KT. ABMR is diagnosed by the reappearance or increase of DSA in serum, combined with typical histopathological signs in kidney biopsy [109]. The principle of treatment is to eliminate existing antibodies and prevent the production of new antibodies. KDIGO guidelines suggest that using TA (PE, IAS) and IVIGs combined with valid immunosuppression (corticosteroids, anti-CD20 antibodies, or lymphocyte-depleting antibodies) could effectively treat ABMR [110].

According to the Banff 2019 Kidney Meeting Report, ABMR has four diagnostic categories, including active ABMR (aABMR), chronic active ABMR (cABMR), chronic inactive ABMR, and C4d staining without evidence of rejection [111]. Active ABMR is severe and results in graft loss, but it is responsive to current treatments [112]. Compared to PE alone or IVIGs alone, PE combined with rituximab and IVIGs showed benefits for aABMR [113]. IAS is also an add-on treatment for ABMR. However, cABMR is the main cause of long-term graft loss, and the treatment remains a challenge. Although PE combined with rituximab and IVIGs has been used in several cABMR cases, there is no clinical improvement either in graft survival or renal functional tests to support the protocol [114,115]. There was no significant difference in the graft survival of 23 patients who were treated with PE and IVIGs combined with rituximab compared to 39 patients who did not receive treatment. The rate of severe infections was also higher in the treated group [114]. Further prospective studies with longer follow-up or innovative therapeutic approaches are necessary.

ABMR is caused by direct and complement-mediated effects of DSA on the allograft [116]. In a recent study using nonantigen-specific IAS, graft survival was 83%, with 100% patient survival after one year [117]. The estimated glomerular filtration rate (eGFR) tended to increase as in other trials [107], which indicated the ability of IAS to delay kidney damage caused by aABMR. For cABMR in this study, eGFR increased by 14.5 mL/min per 1.73 m^2^ after the therapy, which contradicts other studies that reported a decrease in eGFR. This indicates that nonantigen-specific IAS with antihuman Ig-column may have benefits for the treatment of cABMR. No guidelines on the treatment of cABMR are currently available.

A recent study presented that the addition of membrane filtration (MF) in the treatment of patients with severe C4d + humoral rejection who do not respond to IAS can further improve the efficacy of therapy [118]. It can eliminate not only substantial IgG but also macromolecular effector components, such as IgM, C1q, and mannose-binding lectin (MBL). This study suggested that the combination of IAS and MF might be an effective therapy for refractory aABMR.

### Recurrence of kidney diseases after transplantation

Recurrent NS after transplantation is still a challenge; type I membranoproliferative glomerulonephritis (MPGN) and FSGS are the most easily recurrent glomerular diseases. FSGS recurred in more than 50% of patients after KT [119]. PE or IAS is the first-line therapy in patients with recurrent FSGS after KT and can lead to partial or complete remission in over 50% of patients.

According to ASFA guidelines on the use of TA [1], PE should be started once FSGS has recurred, which can improve proteinuria by reducing the concentration of circulatory permeability factors. The overall reported remission rate is 50–70% [1]. In a large systematic review, 71% of patients (*n* = 423) with recurrent FSGS after KT achieved complete or partial remission after treatment with PE [120]. In addition, recurrent patients treated with PE within two weeks had a higher remission rate of proteinuria. Therefore, for transplant recipients with previous diagnose of FSGS, detecting proteinuria routinely after transplantation is important. Once proteinuria reappears, it is recommended to start PE immediately. A review summarized data from 23 studies and found prophylactic PE did not show a reduction in recurrence of FSGS in two thirds of the studies [121].

Reports of using IAS in FSGS are rare. Unselective IAS (mainly using Immunoadsorba^®^ or Globaffin^®^ columns) can be used to reduce the circulatory permeability factors. In a pediatric cohort, 12 children with recurrent FSGS were treated with IAS along with other treatments, including rituximab, abatacept, or galactose. Ten patients responded to the treatment, with two achieving partial remission and eight achieving complete remission [122]. This study has shown the benefit of IAS in recurrent FSGS, but larger prospective, randomized studies are still needed. A combination of IAS and rituximab has been mentioned as an effective treatment in some case reports [123,124]. Thus, PE or IAS might be beneficial for some patients with relapsed and/or refractory FSGS, especially after KT (Table 4). However, which one is more efficacious is unclear, and evidence from randomized controlled trials is still needed.

**Table 4.** Therapeutic apheresis with immunosuppressive agents in recurrent post-transplant focal segmental glomerulosclerosis.

Therapeutic apheresis	Drugs	Subjects	Results	Author [Ref]
PE	–	25	PR (*n* = 11) or CR (*n* = 7)	Uffing A [128]
	+Cyclophosphamide	1	No remission	
	+Rituximab	30	PR (*n* = 9) or CR (*n* = 5)	
IAS	+Rituximab	12 Children	PR (*n* = 2) or CR (*n* = 8) Two maintained remission without IAS and eight became IAS dependent after 3 months.	Allard L [122]
	+Rituximab	7 Patients	PR (*n* = 6) or CR (*n* = 1) 12 months after IAS, allograft survival was 100%	Naciri Bennani H [129]
LDL-A	+Methylprednisolone	7 Children	PR (*n* = 3) or CR (*n* = 4)	Shah L [125]

PE: Plasma exchange; IAS: immunoadsorption; LDL-A: low-density lipoprotein apheresis; PR: partial remission; CR: complete remission.

About 50% of recurrent cases cannot achieve complete remission with PE; thus, additional treatment is needed. In a recent study, seven children with recurrent FSGS after transplantation were treated with LDL-A coupled with methylprednisolone [125]. All patients achieved partial or complete remission with the reduction of urinary protein to creatinine ratios. This novel treatment for recurrent FSGS may be superior to PE, and further randomized studies are needed (Table 4).

About 14–61% of MPGN recur after KT, which is associated with graft loss rates ranging between 14% and 78% [126]. In one case, a 42-year-old woman with recurrent MPGN was treated with PE and showed a progressive reduction in the serum creatinine and proteinuria [127], whereas in another case, a 34-year-old man with recurrent MPGN was treated with cyclophosphamide, oral prednisolone, and PE. However, renal dysfunction progressed, and hemodialysis was restarted. These opposite results show that treatments of recurrent type I MPGN need further investigation.

## Conclusions

TA has been widely used for the treatment of primary or secondary kidney diseases and KT. In LN and KT, IAS seems to be a more favorable extracorporeal therapy compared with PE, but in many other situations, the use of IAS in the treatment of different kidney diseases is still controversial due to the lack of randomized controlled trials to prove the benefits of this method. PE is still the leading extracorporeal therapy in these situations. In addition, PE not only can delete most plasma proteins but also can substitute useful plasma components as needed, such as in the cases of TTP and aHUS. However, PE is increasingly being replaced by DFPP or IAS, which are more selective. IAS provides a choice for the treatment of diseases and reduces life-threatening situations in severe conditions. In the future, IAS may replace PE as the preferred extracorporeal treatment for certain diseases because of its higher efficacy and fewer side effects. For patients with KT or other potential conditions for which long-term treatment is needed, reusable technology may have some advantages because multiple single-use treatments can also increase treatment costs. But in most cases, the considerable costs of IAS still limit the wide use of this technology. There are still many unanswered questions, including optimal timing, dosage, duration of TA, and choice of columns. Further studies to examine and refine the optimal treatment protocols are required.

## References

[1] Padmanabhan A, Connelly-Smith L, Aqui N, et al. Guidelines on the use of therapeutic apheresis in clinical practice – evidence-based approach from the writing committee of the American Society for Apheresis: the eighth special issue. J Clin Apher. 2019;34(3):842–354. 10.1002/jca.21705 31180581

[2] Williams ME, Balogun RA. Principles of separation: indications and therapeutic targets for plasma exchange. Clin J Am Soc Nephrol. 2014;9(1):181–190. 24178973 10.2215/CJN.04680513 PMC3878701

[3] Levy J, Pusey CD. Plasma exchange. In: Floege J, Johnson RJ, Feehally J, editors. Comprehensive clinical nephrology. 4th ed. Philadelphia: Mosby; 2010. p. 1108–1116.

[4] Hafer C, Golla P, Gericke M, et al. Membrane versus centrifuge-based therapeutic plasma exchange: a randomized prospective crossover study. Int Urol Nephrol. 2016;48(1):133–138. 26531062 10.1007/s11255-015-1137-3 PMC5360823

[5] Yamaji K. Immunoadsorption for collagen and rheumatic diseases. Transfusion and Apheresis Science. 2017;56(5):666–670. 28970002 10.1016/j.transci.2017.08.012

[6] Hirano R, Namazuda K, Hirata N. Double filtration plasmapheresis: review of current clinical applications. Ther Apher Dial. 2021;25(2):145–151. 32558286 10.1111/1744-9987.13548

[7] Poullin P, Announ N, Mugnier B, et al. Protein A-immunoadsorption (prosorba column) in the treatment of rheumatoid arthritis. Joint Bone Spine. 2005;72(2):101–103. 15797486 10.1016/j.jbspin.2004.02.009

[8] Raina R, Krishnappa V. An update on LDL apheresis for nephrotic syndrome. Pediatr Nephrol. 2019;34(10):1655–1669. 30218191 10.1007/s00467-018-4061-9

[9] Königshausen E, Sellin L. Circulating permeability factors in primary focal segmental glomerulosclerosis: a review of proposed candidates. Biomed Res Int. 2016;2016:3765608. 27200372 10.1155/2016/3765608 PMC4856884

[10] Ren H, Shen P, Li X, et al. Tacrolimus versus cyclophosphamide in steroid-dependent or steroid-resistant focal segmental glomerulosclerosis: a randomized controlled trial. Am J Nephrol. 2013;37(1):84–90. 23343906 10.1159/000346256

[11] Beer A, Mayer G, Kronbichler A. Treatment strategies of adult primary focal segmental glomerulosclerosis: a systematic review focusing on the last two decades. Biomed Res Int. 2016;2016:4192578. 27144166 10.1155/2016/4192578 PMC4838780

[12] Lau EW, Ma PH, Wu X, et al. Mycophenolate mofetil for primary focal segmental glomerulosclerosis: systematic review. Ren Fail. 2013;35(6):914–929. 23751146 10.3109/0886022X.2013.794687

[13] Hansrivijit P, Cheungpasitporn W, Thongprayoon C, et al. Rituximab therapy for focal segmental glomerulosclerosis and minimal change disease in adults: a systematic review and meta-analysis. BMC Nephrol. 2020;21(1):134. 32293308 10.1186/s12882-020-01797-7 PMC7160971

[14] Korbet SM. The treatment of primary focal segmental glomerulosclerosis. Ren Fail. 2000;22(6):685–696. 11104158 10.1081/jdi-100101956

[15] Mitwalli AH. Adding plasmapheresis to corticosteroids and alkylating agents: does it benefit patients with focal segmental glomerulosclerosis? Nephrol Dial Transplant. 1998;13(6):1524–1528. 9641186 10.1093/ndt/13.6.1524

[16] Moriconi L, Lenti C, Puccini R, et al. Proteinuria in focal segmental glomerulosclerosis: role of circulating factors and therapeutic approach. Ren Fail. 2001;23(3–4):533–541. 11499567 10.1081/jdi-100104735

[17] Muso E, Mune M, Hirano T, et al. A prospective observational survey on the long-term effect of LDL apheresis on drug-resistant nephrotic syndrome. Nephron Extra. 2015;5(2):58–66. 26557843 10.1159/000437338 PMC4592509

[18] Feld SM, Figueroa P, Savin V, et al. Plasmapheresis in the treatment of steroid-resistant focal segmental glomerulosclerosis in native kidneys. Am J Kidney Dis. 1998;32(2):230–237. 9708606 10.1053/ajkd.1998.v32.pm9708606

[19] Beck LH, Jr., Bonegio RG, Lambeau G, et al. M-type phospholipase A2 receptor as target antigen in idiopathic membranous nephropathy. N Engl J Med. 2009;361(1):11–21. 19571279 10.1056/NEJMoa0810457 PMC2762083

[20] Tomas NM, Beck LH, Jr., Meyer-Schwesinger C, et al. Thrombospondin type-1 domain-containing 7A in idiopathic membranous nephropathy. N Engl J Med. 2014;371(24):2277–2287. 25394321 10.1056/NEJMoa1409354 PMC4278759

[21] Sethi S, Debiec H, Madden B, et al. Neural epidermal growth factor-like 1 protein (NELL-1) associated membranous nephropathy. Kidney Int. 2020;97(1):163–174. 31901340 10.1016/j.kint.2019.09.014

[22] Sethi S, Madden BJ, Debiec H, et al. Exostosin 1/exostosin 2-associated membranous nephropathy. J Am Soc Nephrol. 2019;30(6):1123–1136. 31061139 10.1681/ASN.2018080852 PMC6551791

[23] Ponticelli C, Zucchelli P, Passerini P, et al. A 10-year follow-up of a randomized study with methylprednisolone and chlorambucil in membranous nephropathy. Kidney Int. 1995;48(5):1600–1604. 8544420 10.1038/ki.1995.453

[24] Cattran DC, Appel GB, Hebert LA, et al. Cyclosporine in patients with steroid-resistant membranous nephropathy: a randomized trial. Kidney Int. 2001;59(4):1484–1490. 11260412 10.1046/j.1523-1755.2001.0590041484.x

[25] Beck L, Bomback AS, Choi MJ, et al. KDOQI US commentary on the 2012 KDIGO clinical practice guideline for glomerulonephritis. Am J Kidney Dis. 2013;62(3):403–441. 23871408 10.1053/j.ajkd.2013.06.002

[26] Perna A, Schieppati A, Zamora J, et al. Immunosuppressive treatment for idiopathic membranous nephropathy: a systematic review. Am J Kidney Dis. 2004;44(3):385–401. 15332211

[27] Müller-Deile J, Schiffer L, Hiss M, et al. A new rescue regimen with plasma exchange and rituximab in high-risk membranous glomerulonephritis. Eur J Clin Invest. 2015;45(12):1260–1269. 26444294 10.1111/eci.12545

[28] Esnault VL, Besnier D, Testa A, et al. Effect of protein a immunoadsorption in nephrotic syndrome of various etiologies. J Am Soc Nephrol. 1999;10(9):2014–2017. 10477155 10.1681/ASN.V1092014

[29] Hamilton P, Kanigicherla D, Hanumapura P, et al. Peptide GAM immunoadsorption therapy in primary membranous nephropathy (PRISM): phase II trial investigating the safety and feasibility of peptide GAM immunoadsorption in anti-PLA2 R positive primary membranous nephropathy. J Clin Apher. 2018;33(3):283–290. 29094388 10.1002/jca.21599

[30] Eskandary F, Wahrmann M, Biesenbach P, et al. ABO antibody and complement depletion by immunoadsorption combined with membrane filtration–a randomized, controlled, cross-over trial. Nephrol Dial Transplant. 2014;29(3):706–714. 24378525 10.1093/ndt/gft502

[31] Weinmann-Menke J, Holtz S, Sollinger D, et al. Treatment of membranous nephropathy in patients with THSD7A antibodies using immunoadsorption. Am J Kidney Dis. 2019;74(6):849–852. 31451329 10.1053/j.ajkd.2019.05.021

[32] Dammacco F, Battaglia S, Gesualdo L, et al. Goodpasture's disease: a report of ten cases and a review of the literature. Autoimmun Rev. 2013;12(11):1101–1108. 23806563 10.1016/j.autrev.2013.06.014

[33] Kalluri R, Wilson CB, Weber M, et al. Identification of the alpha 3 chain of type IV collagen as the common autoantigen in antibasement membrane disease and goodpasture syndrome. J Am Soc Nephrol. 1995;6(4):1178–1185. 8589284 10.1681/ASN.V641178

[34] Levy JB, Hammad T, Coulthart A, et al. Clinical features and outcome of patients with both ANCA and anti-GBM antibodies. Kidney Int. 2004;66(4):1535–1540. 15458448 10.1111/j.1523-1755.2004.00917.x

[35] Clark WF, Huang S-HS, Walsh MW, et al. Plasmapheresis for the treatment of kidney diseases. Kidney Int. 2016;90(5):974–984. 27498206 10.1016/j.kint.2016.06.009

[36] Lockwood CM, Boulton-Jones JM, Lowenthal RM, et al. Recovery from goodpasture's syndrome after immunosuppressive treatment and plasmapheresis. Br Med J. 1975;2(5965):252–254. 1131574 10.1136/bmj.2.5965.252 PMC1673280

[37] Hu W, Liu Z, Ji D, et al. Staphylococcal protein a immunoadsorption for goodpasture's syndrome in four chinese patients. Journal of Nephrology. 2006;19(3):312–317. 16874690

[38] Laczika K, Knapp S, Derfler K, et al. Immunoadsorption in goodpasture's syndrome. Am J Kidney Dis. 2000;36(2):392–395. 10922318 10.1053/ajkd.2000.8993

[39] Biesenbach P, Kain R, Derfler K, et al. Long-term outcome of anti-glomerular basement membrane antibody disease treated with immunoadsorption. PLoS One. 2014;9(7):e103568. 25079220 10.1371/journal.pone.0103568 PMC4117516

[40] Zhang Y-y, Tang Z, Chen D-M, et al. Comparison of double filtration plasmapheresis with immunoadsorption therapy in patients with anti-glomerular basement membrane nephritis. BMC Nephrol. 2014;15:128. 25086644 10.1186/1471-2369-15-128 PMC4127070

[41] Nakazawa D, Masuda S, Tomaru U, et al. Pathogenesis and therapeutic interventions for ANCA-associated vasculitis. Nat Rev Rheumatol. 2019;15(2):91–101. 30542206 10.1038/s41584-018-0145-y

[42] Yang JJ, Jennette JC, Falk RJ. Immune complex glomerulonephritis is induced in rats immunized with heterologous myeloperoxidase. Clin Exp Immunol. 1994;97(3):466–473. 8082301 10.1111/j.1365-2249.1994.tb06111.x PMC1534869

[43] Jayne DR, Gaskin G, Rasmussen N, et al. Randomized trial of plasma exchange or high-dosage methylprednisolone as adjunctive therapy for severe renal vasculitis. J Am Soc Nephrol. 2007;18(7):2180–2188. 17582159 10.1681/ASN.2007010090

[44] Kronbichler A, Shin JI, Wang C-S, et al. Plasma exchange in ANCA-associated vasculitis: the pro position. Nephrology, dialysis, transplantation: official publication of the European Dialysis and Transplant Association. European Renal Association. 2021;36(2):227–231. 10.1093/ndt/gfaa311 33374018

[45] Walsh M, Merkel PA, Peh CA, et al. Plasma exchange and glucocorticoids in severe ANCA-associated vasculitis. N Engl J Med. 2020;382(7):622–631. 32053298 10.1056/NEJMoa1803537 PMC7325726

[46] Bellos I, Michelakis I, Nikolopoulos D. The role of plasma exchange in antineutrophil cytoplasmic antibody-associated vasculitis: a meta-analysis. Clin Rheumatol. 2021;40(4):1447–1456. 32935248 10.1007/s10067-020-05390-z

[47] Kidney Disease: Improving Global Outcomes (KDIGO) Glomerular Diseases Work Group. KDIGO 2021 clinical practice guideline for the management of glomerular diseases. Kidney Int. 2021;100(4S):S1–S276. 34556256 10.1016/j.kint.2021.05.021

[48] Palmer A, Cairns T, Dische F, et al. Treatment of rapidly progressive glomerulonephritis by extracorporeal immunoadsorption, prednisolone and cyclophosphamide. Nephrol Dial Transplant. 1991;6(8):536–542. 1956551 10.1093/ndt/6.8.536

[49] C P, WF C. 2015. Canadian Apheresis Group 2014 plasma exchange data review: hematological, renal/collagen vascular, dermatological and transplant. Data review. Paper presented at: 35th Annual General Meeting of the Canadian Apheresis Group.

[50] Brocklebank V, Wood KM, Kavanagh D. Thrombotic microangiopathy and the kidney. Clin J Am Soc Nephrol. 2018;13(2):300–317. 29042465 10.2215/CJN.00620117 PMC5967417

[51] Soejima K, Mimura N, Hirashima M, et al. A novel human metalloprotease synthesized in the liver and secreted into the blood: possibly, the von willebrand factor-cleaving protease? J Biochem. 2001;130(4):475–480. 11574066 10.1093/oxfordjournals.jbchem.a003009

[52] Fujimura Y, Matsumoto M. Registry of 919 patients with thrombotic microangiopathies across Japan: database of Nara medical university during 1998-2008. Intern Med. 2010;49(1):7–15. 20045995 10.2169/internalmedicine.49.2706

[53] Scully M, Hunt BJ, Benjamin S, et al. Guidelines on the diagnosis and management of thrombotic thrombocytopenic purpura and other thrombotic microangiopathies. Br J Haematol. 2012;158(3):323–335. 22624596 10.1111/j.1365-2141.2012.09167.x

[54] Sarode R, Bandarenko N, Brecher ME, et al. Thrombotic thrombocytopenic purpura: 2012 American Society for Apheresis (ASFA) consensus conference on classification, diagnosis, management, and future research. J Clin Apher. 2014;29(3):148–167. 24136342 10.1002/jca.21302

[55] Rock GA, Shumak KH, Buskard NA, et al. Comparison of plasma exchange with plasma infusion in the treatment of thrombotic thrombocytopenic purpura. Canadian Apheresis Study Group. N Engl J Med. 1991;325(6):393–397. 2062330 10.1056/NEJM199108083250604

[56] Cataland SR, Kourlas PJ, Yang S, et al. Cyclosporine or steroids as an adjunct to plasma exchange in the treatment of immune-mediated thrombotic thrombocytopenic purpura. Blood Adv. 2017;1(23):2075–2082. 29296854 10.1182/bloodadvances.2017009308 PMC5728286

[57] Dane K, Chaturvedi S. Beyond plasma exchange: novel therapies for thrombotic thrombocytopenic purpura. Hematology Am Soc Hematol Educ Program. 2018;2018(1):539–547. 30504355 10.1182/asheducation-2018.1.539 PMC6246029

[58] Knoebl P, Cataland S, Peyvandi F, et al. Efficacy and safety of open-label caplacizumab in patients with exacerbations of acquired thrombotic thrombocytopenic purpura in the HERCULES study. J Thromb Haemost. 2020;18(2):479–484. 31691462 10.1111/jth.14679 PMC7027866

[59] Sukumar S, George JN, Cataland SR. Shared decision making, thrombotic thrombocytopenic purpura, and caplacizumab. Am J Hematol. 2020;95(4):E76–e77. 31894592 10.1002/ajh.25715 PMC9292479

[60] van Dorland HA, Taleghani MM, Sakai K, et al. The international hereditary thrombotic thrombocytopenic purpura registry: key findings at enrollment until 2017. Haematologica. 2019;104(10):2107–2115. 30792199 10.3324/haematol.2019.216796 PMC6886414

[61] Knöbl P, Haas M, Laczika K, et al. Immunoadsorption for the treatment of a patient with severe thrombotic thrombocytopenic purpura resistant to plasma exchange: kinetics of an inhibitor of ADAMTS13. J Thromb Haemost. 2003;1(1):187–189. 12871558 10.1046/j.1538-7836.2003.00004.x

[62] Drew MJ. Resolution of refractory, classic thrombotic thrombocytopenic purpura after staphylococcal protein a immunoadsorption. Transfusion. 1994;34(6):536–538. 8023397 10.1046/j.1537-2995.1994.34694295072.x

[63] Tarr PI, Gordon CA, Chandler WL. Shiga-toxin-producing *Escherichia coli* and haemolytic uraemic syndrome. Lancet. 2005;365(9464):1073–1086. 15781103 10.1016/S0140-6736(05)71144-2

[64] Greinacher A, Friesecke S, Abel P, et al. Treatment of severe neurological deficits with IgG depletion through immunoadsorption in patients with Escherichia coli O104:H4-associated haemolytic uraemic syndrome: a prospective trial. Lancet. 2011;378(9797):1166–1173. 21890192 10.1016/S0140-6736(11)61253-1

[65] Noris M, Remuzzi G. Atypical hemolytic-uremic syndrome. N Engl J Med. 2009;361(17):1676–1687. 19846853 10.1056/NEJMra0902814

[66] Frémeaux-Bacchi V, Miller EC, Liszewski MK, et al. Mutations in complement C3 predispose to development of atypical hemolytic uremic syndrome. Blood. 2008;112(13):4948–4952. 18796626 10.1182/blood-2008-01-133702 PMC2597601

[67] Delvaeye M, Noris M, De Vriese A, et al. Thrombomodulin mutations in atypical hemolytic-uremic syndrome. N Engl J Med. 2009;361(4):345–357. 19625716 10.1056/NEJMoa0810739 PMC3530919

[68] Józsi M, Licht C, Strobel S, et al. Factor H autoantibodies in atypical hemolytic uremic syndrome correlate with CFHR1/CFHR3 deficiency. Blood. 2008;111(3):1512–1514. 18006700 10.1182/blood-2007-09-109876

[69] Bayer G, von Tokarski F, Thoreau B, et al. Etiology and outcomes of thrombotic microangiopathies. CJASN. 2019;14(4):557–566. 30862697 10.2215/CJN.11470918 PMC6450353

[70] Fakhouri F, Hourmant M, Campistol JM, et al. Terminal complement inhibitor eculizumab in adult patients with atypical hemolytic uremic syndrome: a Single-Arm, Open-Label trial. Am J Kidney Dis. 2016;68(1):84–93. 27012908 10.1053/j.ajkd.2015.12.034

[71] Noris M, Caprioli J, Bresin E, et al. Relative role of genetic complement abnormalities in sporadic and familial aHUS and their impact on clinical phenotype. Clin J Am Soc Nephrol. 2010;5(10):1844–1859. 20595690 10.2215/CJN.02210310 PMC2974386

[72] Cao M, Leite BN, Ferreiro T, et al. Eculizumab modifies outcomes in adults with atypical hemolytic uremic syndrome with acute kidney injury. Am J Nephrol. 2018;48(3):225–233. 30205388 10.1159/000492865

[73] Mannik M, Merrill CE, Stamps LD, et al. Multiple autoantibodies form the glomerular immune deposits in patients with systemic lupus erythematosus. J Rheumatol. 2003;30(7):1495–1504. 12858447

[74] Hohenstein B, Bornstein SR, Aringer M. Immunoadsorption for connective tissue disease. Atheroscler Suppl. 2013;14(1):185–189. 23357163 10.1016/j.atherosclerosissup.2012.10.034

[75] Illei GG, Austin HA, Crane M, et al. Combination therapy with pulse cyclophosphamide plus pulse methylprednisolone improves long-term renal outcome without adding toxicity in patients with lupus nephritis. Ann Intern Med. 2001;135(4):248–257. 11511139 10.7326/0003-4819-135-4-200108210-00009

[76] Jones JV, Cumming RH, Bucknall RC, et al. Plasmapheresis in the management of acute systemic lupus erythematosus? Lancet. 1976;1(7962):709–711. 56531 10.1016/s0140-6736(76)93088-9

[77] Lewis EJ, Hunsicker LG, Lan SP, et al. A controlled trial of plasmapheresis therapy in severe lupus nephritis. The lupus nephritis collaborative study group. N Engl J Med. 1992;326(21):1373–1379. 1569973 10.1056/NEJM199205213262101

[78] Aringer M, Smolen JS, Graninger WB. Severe infections in plasmapheresis-treated systemic lupus erythematosus. Arthritis Rheumat. 1998;41(3):414–420. 9506568 10.1002/1529-0131(199803)41:3<414::AID-ART6>3.0.CO;2-N

[79] Bertsias GK, Tektonidou M, Amoura Z, et al. Joint European League Against Rheumatism and European Renal Association-European Dialysis and Transplant Association (EULAR/ERA-EDTA) recommendations for the management of adult and paediatric lupus nephritis. Ann Rheum Dis. 2012;71(11):1771–1782. 22851469 10.1136/annrheumdis-2012-201940 PMC3465859

[80] Soyuöz A, Karadağ Ö, Karaağaç T, et al. Therapeutic plasma exchange for refractory SLE: a comparison of outcomes between different sub-phenotypes. Eur J Rheumatol. 2018;5(1):32–36. 29657872 10.5152/eurjrheum.2017.17088 PMC5895148

[81] Stummvoll G, Aringer M, Handisurya A, et al. Immunoadsorption in autoimmune diseases affecting the kidney. Semin Nephrol. 2017;37(5):478–487. 28863794 10.1016/j.semnephrol.2017.05.020

[82] Stummvoll GH, Aringer M, Smolen JS, et al. IgG immunoadsorption reduces systemic lupus erythematosus activity and proteinuria: a long term observational study. Ann Rheum Dis. 2005;64(7):1015–1021. 15640267 10.1136/ard.2004.029660 PMC1755551

[83] Gaubitz M, Seidel M, Kummer S, et al. Prospective randomized trial of two different immunoadsorbers in severe systemic lupus erythematosus. J Autoimmun. 1998;11(5):495–501. 9802935 10.1006/jaut.1998.0229

[84] Stummvoll GH, Aringer M, Jansen M, et al. Immunoadsorption (IAS) as a rescue therapy in SLE: considerations on safety and efficacy. Wien Klin Wochenschr. 2004;116(21-22):716–724. 15628641 10.1007/s00508-004-0232-8

[85] Stummvoll GH, Schmaldienst S, Smolen JS, et al. Lupus nephritis: prolonged immunoadsorption (IAS) reduces proteinuria and stabilizes global disease activity. Nephrol Dial Transplant. 2012;27(2):618–626. 21617196 10.1093/ndt/gfr239

[86] Xu L, Wu X, Zou Y. Clinical efficacy comparison of HA280 and DNA280 immunoadsorption column in treating systemic lupus erythematosus. Modern Rheumatology. 2016;26(1):94–98. 26025438 10.3109/14397595.2015.1056955

[87] Segev DL, Kucirka LM, Gentry SE, et al. Utilization and outcomes of kidney paired donation in the United States. Transplantation. 2008;86(4):502–510. 18724216 10.1097/TP.0b013e3181812f85

[88] Egawa H, Tanabe K, Fukushima N, et al. Current status of organ transplantation in Japan. Am J Transplant. 2012;12(3):523–530. 22054061 10.1111/j.1600-6143.2011.03822.x

[89] Becker LE, Süsal C, Morath C. Kidney transplantation across HLA and ABO antibody barriers. Curr Opin Organ Transplant. 2013;18(4):445–454. 23838650 10.1097/MOT.0b013e3283636c20

[90] Muramatsu M, Gonzalez HD, Cacciola R, et al. ABO incompatible renal transplants: good or bad? World J Transplant. 2014;4(1):18–29. 24669364 10.5500/wjt.v4.i1.18 PMC3964193

[91] Alexandre GP, Squifflet JP, Bruyère D, et al. Present experiences in a series of 26 ABO-incompatible living donor renal allografts. Transpl Proc. 1987;19(6):4538–4542. 3321614

[92] Sawada T, Fuchinoue S, Teraoka S. Successful A1-to-O ABO-incompatible kidney transplantation after a preconditioning regimen consisting of anti-CD20 monoclonal antibody infusions, splenectomy, and double-filtration plasmapheresis. Transplantation. 2002;74(9):1207–1210. 12451255 10.1097/00007890-200211150-00001

[93] Takahashi K, Saito K. ABO-incompatible kidney transplantation. Transplant Rev. 2013;27(1):1–8. 10.1016/j.trre.2012.07.003 22902167

[94] Tanabe K, Ishida H, Inui M, et al. ABO-incompatible kidney transplantation: long-term outcomes. In: Terasaki PI, Cecka JM, editors. Clinical transplants. Los Angeles: Terasaki Foundation Lab; 2013. p. 307–312. 25095522

[95] Takahashi K, Saito K, Takahara S, et al. Excellent long-term outcome of ABO-incompatible living donor kidney transplantation in Japan. Am J Transplant. 2004;4(7):1089–1096. 15196066 10.1111/j.1600-6143.2004.00464.x

[96] Tiwari AK, Aggarwal G, Arora D, et al. Immunoadsorption in ABO-incompatible kidney transplantation in adult and pediatric patients with follow-up on graft and patient survival: first series from India. Asian J Transfus Sci. 2020;14(1):13–18. 33162699 10.4103/ajts.AJTS_82_19 PMC7607981

[97] Lo P, Sharma A, Craig JC, et al. Preconditioning therapy in ABO-incompatible living kidney transplantation: a systematic review and meta-analysis. Transplantation. 2016;100(4):933–942. 26425876 10.1097/TP.0000000000000933

[98] Barnett AN, Manook M, Nagendran M, et al. Tailored desensitization strategies in ABO blood group antibody incompatible renal transplantation. Transpl Int. 2014;27(2):187–196. 24188566 10.1111/tri.12234

[99] Yeh JH, Chiu HC. Coagulation abnormalities in serial double-filtration plasmapheresis. J Clin Apheresis. 2001;16(3):139–142. 11746541 10.1002/jca.1026

[100] Sethi S, Choi J, Toyoda M, et al. Desensitization: overcoming the immunologic barriers to transplantation. J Immunol Res. 2017;2017:6804678. 28127571 10.1155/2017/6804678 PMC5239985

[101] Pruthi R, Hilton R, Pankhurst L, et al. UK renal registry 16th annual report: chapter 4 demography of patients waitlisted for renal transplantation in the UK: national and centre-specific analyses. Nephron Clin Pract. 2013;125(1-4):81–98. 24662168 10.1159/000360023

[102] Loupy A, Suberbielle-Boissel C, Zuber J, et al. Combined posttransplant prophylactic IVIg/anti-CD 20/plasmapheresis in kidney recipients with preformed donor-specific antibodies: a pilot study. Transplantation. 2010;89(11):1403–1410. 20386362 10.1097/TP.0b013e3181da1cc3

[103] Cardella CJ, Sutton D, Uldall PR, et al. Intensive plasma exchange and renal-transplant rejection. Lancet. 1977;1(8005):264. 10.1016/s0140-6736(77)91069-8 64800

[104] Stegall MD, Gloor J, Winters JL, et al. A comparison of plasmapheresis versus high-dose IVIG desensitization in renal allograft recipients with high levels of donor specific alloantibody. Am J Transplant. 2006;6(2):346–351. 16426319 10.1111/j.1600-6143.2005.01178.x

[105] Taube DH, Williams DG, Cameron JS, et al. Renal transplantation after removal and prevention of resynthesis of HLA antibodies. Lancet. 1984;1(8381):824–828. 6143141 10.1016/s0140-6736(84)92273-6

[106] Palmer A, Taube D, Welsh K, et al. Removal of anti-HLA antibodies by extracorporeal immunoadsorption to enable renal transplantation. Lancet. 1989;1(8628):10–12. 2563001 10.1016/s0140-6736(89)91672-3

[107] Böhmig GA, Wahrmann M, Regele H, et al. Immunoadsorption in severe C4d-positive acute kidney allograft rejection: a randomized controlled trial. Am J Transplant. 2007;7(1):117–121. 17109725 10.1111/j.1600-6143.2006.01613.x

[108] Noble J, Metzger A, Naciri Bennani H, et al. Apheresis efficacy and tolerance in the setting of HLA-incompatible kidney transplantation. JCM. 2021;10(6):1316. 33806743 10.3390/jcm10061316 PMC8005077

[109] Sis B, Mengel M, Haas M, et al. Banff '09 meeting report: antibody mediated graft deterioration and implementation of Banff working groups. Am J Transplant. 2010;10(3):464–471. 20121738 10.1111/j.1600-6143.2009.02987.x

[110] Chadban SJ, Ahn C, Axelrod DA, et al. Summary of the kidney disease: improving global outcomes (KDIGO) clinical practice guideline on the evaluation and management of candidates for kidney transplantation. Transplantation. 2020;104(4):708–714. 32224812 10.1097/TP.0000000000003137 PMC7147399

[111] Loupy A, Haas M, Roufosse C, et al. The Banff 2019 kidney meeting report (I): updates on and clarification of criteria for T cell- and antibody-mediated rejection. Am J Transplant. 2020;20(9):2318–2331. 32463180 10.1111/ajt.15898 PMC7496245

[112] Pape L, Becker JU, Immenschuh S, et al. Acute and chronic antibody-mediated rejection in pediatric kidney transplantation. Pediatr Nephrol. 2015;30(3):417–424. 24865478 10.1007/s00467-014-2851-2

[113] Xie P, Tao M, Peng K, et al. Plasmapheresis therapy in kidney transplant rejection. Blood Purif. 2019;47(1–3):73–84. 30253400 10.1159/000493521

[114] Piñeiro GJ, De Sousa-Amorim E, Solé M, et al. Rituximab, plasma exchange and immunoglobulins: an ineffective treatment for chronic active antibody-mediated rejection. BMC Nephrol. 2018;19(1):261. 30309322 10.1186/s12882-018-1057-4 PMC6182805

[115] Mella A, Gallo E, Messina M, et al. Treatment with plasmapheresis, immunoglobulins and rituximab for chronic-active antibody-mediated rejection in kidney transplantation: clinical, immunological and pathological results. World J Transplant. 2018;8(5):178–187. 30211026 10.5500/wjt.v8.i5.178 PMC6134268

[116] Gloor J, Cosio F, Lager DJ, et al. The spectrum of antibody-mediated renal allograft injury: implications for treatment. Am J Transplant. 2008;8(7):1367–1373. 18510643 10.1111/j.1600-6143.2008.02262.x

[117] Montagud-Marrahi E, Revuelta I, Cucchiari D, et al. Successful use of nonantigen-specific immunoadsorption with antihuman Ig-columns in kidney graft antibody-mediated rejection. J Clin Apher. 2020;35(3):188–199. 32219886 10.1002/jca.21779

[118] Doberer K, Bond G, Kläger J, et al. Immunoadsorption combined with membrane filtration to counteract early treatment-refractory antibody-mediated rejection. Blood Purif. 2020;49(5):576–585. 32191942 10.1159/000506504

[119] D'Agati VD, Kaskel FJ, Falk RJ. Focal segmental glomerulosclerosis. N Engl J Med. 2011;365(25):2398–2411. 22187987 10.1056/NEJMra1106556

[120] Kashgary A, Sontrop JM, Li L, et al. The role of plasma exchange in treating post-transplant focal segmental glomerulosclerosis: a systematic review and meta-analysis of 77 case-reports and case-series. BMC Nephrol. 2016;17(1):104. 27473582 10.1186/s12882-016-0322-7 PMC4966699

[121] Al Shamsi HR, Shaheen I, Aziz D. Management of recurrent focal segmental glomerulosclerosis (FSGS) post renal transplantation. Transplant Rev. 2022;36(1):100675. 10.1016/j.trre.2021.100675 34952298

[122] Allard L, Kwon T, Krid S, et al. Treatment by immunoadsorption for recurrent focal segmental glomerulosclerosis after paediatric kidney transplantation: a multicentre french cohort. Nephrol Dial Transplant. 2018;33(6):954–963. 28992235 10.1093/ndt/gfx214

[123] Martin-Moreno PL, Rifon J, Errasti P. Efficacy of the combination of immunoadsorption and rituximab for treatment in a case of severe focal and segmental glomerulosclerosis recurrence after renal transplantation. Blood Purif. 2018;46(2):90–93. 29672314 10.1159/000488638

[124] Meyer TN, Thaiss F, Stahl RA. Immunoadsorbtion and rituximab therapy in a second living-related kidney transplant patient with recurrent focal segmental glomerulosclerosis. Transpl Int. 2007;20(12):1066–1071. 17887957 10.1111/j.1432-2277.2007.00562.x

[125] Shah L, Hooper DK, Okamura D, et al. LDL-apheresis-induced remission of focal segmental glomerulosclerosis recurrence in pediatric renal transplant recipients. Pediatr Nephrol. 2019;34(11):2343–2350. 31250206 10.1007/s00467-019-04296-6

[126] Green H, Rahamimov R, Rozen-Zvi B, et al. Recurrent membranoproliferative glomerulonephritis type I after kidney transplantation: a 17-year single-center experience. Transplantation. 2015;99(6):1172–1177. 25340602 10.1097/TP.0000000000000459

[127] Saxena R, Frankel WL, Sedmak DD, et al. Recurrent type I membranoproliferative glomerulonephritis in a renal allograft: successful treatment with plasmapheresis. Am J Kidney Dis. 2000;35(4):749–752. 10739799 10.1016/s0272-6386(00)70025-4

[128] Uffing A, Pérez-Sáez MJ, Mazzali M, et al. Recurrence of FSGS after kidney transplantation in adults. Clin J Am Soc Nephrol. 2020;15(2):247–256. 31974287 10.2215/CJN.08970719 PMC7015092

[129] Naciri Bennani H, Bonzi JY, Noble J, et al. Immunoadsorption for recurrent primary focal segmental glomerulosclerosis on kidney allografts: a single-center experience and literature review. Blood Purif. 2020;49(3):322–333. 31913143 10.1159/000504244

